# Dynamic change of bacterial diversity, metabolic pathways, and flavor during ripening of the Chinese fermented sausage

**DOI:** 10.3389/fmicb.2022.990606

**Published:** 2022-10-04

**Authors:** Ji Wang, Tariq Aziz, Ruxue Bai, Xin Zhang, Muhammad Shahzad, Manal Y. Sameeh, Ayaz Ali Khan, Anas S. Dablool, Yingchun Zhu

**Affiliations:** ^1^College of Food Science and Engineering, Shanxi Agricultural University, Jinzhong, China; ^2^Pak-Austria Fachhochschule: Institute of Applied Sciences and Technology, Haripur, Pakistan; ^3^School of Biological Sciences, Health and Life Sciences Building, University of Reading, Reading, United Kingdom; ^4^Chemistry Department, Faculty of Applied Sciences, Al-Leith University College, Umm Al-Qura University, Makkah, Saudi Arabia; ^5^Department of Biotechnology, University of Malakand, Chakdara, Pakistan; ^6^Department of Public Health, Health Sciences College Al-Leith, Umm Al-Qura University, Makkah, Saudi Arabia

**Keywords:** fermented sausage, Lp MSZ2, Sx YCC3, bacterial diversity, metabolic pathways, flavor substances

## Abstract

Chinese fermented sausage is a famous fermented meat product with a complex microbiota that has a potential impact on flavor and quality. In this study, *Lactobacillus plantarum* MSZ2 and *Staphylococcus xylosus* YCC3 were used as starter cultures to investigate the change in bacterial diversity, metabolic pathways, and flavor compounds during the ripening process of fermented sausages. High-throughput sequencing technology and headspace solid-phase microextraction-gas chromatography–mass spectrometry (HS-SPME-GC/MS) were applied for characterizing the profiles of bacterial diversity, metabolic pathways, and flavor compounds in sausage samples on days 0, 6, and 12 during ripening. Results showed that *Lactobacillus*, *Staphylococcus*, *Lactococcus*, *Leuconostoc*, and *Weissella* were the most abundant bacterial genera found in the sausage samples during all stages of fermentation. Functional prediction reveals the abundance of 12 different metabolic pathways, the most important pathways are carbohydrate metabolism, nucleotide metabolism, lipid metabolism, and amino acid metabolism. A total of 63 volatile compounds were successfully identified in fermented sausage samples. Correlational analysis demonstrated that *Staphylococcus* and *Leuconostoc* were closely related to the formation of flavor compounds. Therefore, the present study may provide guidance for future use of microbiota to improve flavor, quality, and preservation of fermented sausages.

## Introduction

In China, the most common fermented meat products are sausages, ham, and fish (Wang et al., 2022). Of these, fermented sausages are favored by many Chinese consumers owing to their unique taste and aroma (Wang et al., 1995; Huang et al., 2021). They are prepared from fresh meat, spices, salt, sugar, and other auxiliary materials, inoculated with or without starter culture, and are fermented under specific temperature and humidity conditions either by the action of enzymes or microorganisms (Liu et al., 2020). Historically, fermented sausage is deeply embedded Chinese culture wherein the recipes are passed from one generation to the next as part of food fermentation culture. As an effective preservation strategy, fermentation process not only enhances the shelf life of the fermented meat products but also improves sensory properties, taste, and nutritional value and reduces toxins and food spoilage factors (Blandino et al., 2003; Liu et al., 2011; Hu et al., 2020b).

A variety of microorganisms play a crucial role in different characteristics (including flavor and texture) of fermented meat products during the production and storage process (Wang Z. et al., 2021). These primary microorganisms, such as *Lactic acid bacteria* and *Staphylococcus,* come from different sources including the sausage raw material, inoculation starter cultures, the equipment, and the processing environment (Hu et al., 2020a). These microorganisms help to break down the biochemical compounds of raw materials such as lipids, proteins, and carbohydrates, thus enhancing digestibility, flavor, and the nutritional value of the products (Tamang et al., 2015). Similarly, the traditional fermented sausages are prepared by spontaneous fermentation without the starter culture, which involve complex microbial interactions and metabolic activities (Sarter et al., 2015). The microbial diversity and interactions in the sausages help mainly in lipolysis and proteolysis that giving the final product characteristic taste, quality, nutrition, and sensory properties (Padilla et al., 2014). *Lactobacillus plantarum* and *Staphylococcus xylosus* could secrete lipase and protease, which promote the hydrolysis of lipids and proteins (Wang J. et al., 2020). Other ingredients like sugar, salt, and spices also contribute to the formation of flavor compounds.

To ensure the quality and safety of the fermented sausages and standardize the production process, it is a common practice to use exogenous microorganisms as starter cultures. In most of the cases, the starter cultures composed of Lactic acid bacteria (*LAB*), coagulase-negative cocci, and yeast (Cocolin et al., 2011; Feng et al., 2021). *LAB* such as *Lactiplantibacillus plantarum, Pediococcus pentosaceus,* and *Lactobacillus casei* has long been used as starter culture in fermented sausages. *LAB* bacteria primarily reduce the pH of the sausages through the production of lactic acid. A low pH helps the fibrillar proteins to coagulate, thus increasing the firmness and cohesiveness of the final product. As a result, the sausage is easy to cut and slice (Drosinos et al., 2007). Research evidence suggests that *LAB* strains utilize carbohydrates metabolites (acetic acid, formic acid, and succinic acid) produced by the fermentation process to enhance the flavor (Montel et al., 1998; Xiao et al., 2021). In addition to LAB, coagulase-negative cocci (CNC), such as *Staphylococcus xylosus, Staphylococcus saprophyticus*, and *Staphylococcus carnosus* are also added as starter cultures to manufacture fermented sausages (Mejri et al., 2016). Strong lipolytic and proteolytic activity in *Staphylococci* results in the breakdown of lipids and proteins into smaller molecules and the production of acids, aldehydes, ketones, esters, and alcohols. Their role is not only crucial in flavor formation (Wang Y. et al., 2021), but they also contribute to the color formation of fermented meat products due to the nitrate reductase activity. Some studies by Li et al. (2016) and Ge et al. (2019) demonstrated that these bacterial strains can inhibit the growth of spoilage microorganisms and improve the color, quality, and sensory characteristics of the fermented sausages. Apart from *LAB* and CNC, the fermented meat product including sausages harbor more diverse microflora, the characterization of which has become a hot topic of research in recent years.

Recently, with the development of molecular biological techniques, a new culture-independent method-high-throughput sequencing technology based on DNA sequencing technologies has been widely used to assess microbial community structure and composition (Sun et al., 2018; Zhang et al., 2021). Compared to culture-dependent methods, this technology targets one or more hypervariable regions of the 16S rRNA gene and makes the microbial analysis more comprehensive and cost-effective (Bassey et al., 2021). Data from high-throughput sequencing combined with analytical methods such as Principal Component Analysis (PCA) to analyze the microbial composition. However, these analyses mainly focused on the microbial community, and further studies are needed to analyze the metabolic function of the microbial community in fermented sausages. PICRUSt (phylogenetic investigation of communities by reconstruction of unobserved states) is based on 16S rRNA sequencing data to predict the function of a metagenome through marker gene data and reference genome databases (Langille et al., 2013). Park et al. (2020) utilized PICRUSt to examine the metabolic flux during fermentation and to determine why the system performed better by analyzing functional genes. So far, studies analyzing the correlation between metabolic pathways and the formation of flavor compounds in fermented sausages are limited.

Based on previous studies, we investigated the effect of *L. plantarum* MSZ2 (*Lp* MSZ2) or/and *Staphylococcus xylosus* YCC3 (*Sx* YCC3) on the final fermented sausage, including pH, lipid oxidation, and bacteria count (Wang et al., 2022). The current study used a mixture of *L. plantarum* MSZ2 (*Lp* MSZ2) and *Staphylococcus xylosus* YCC3 (*Sx* YCC3) to dissect bacterial community composition, metabolic pathways, and its role in the production of flavoring compounds during the ripening process of fermented sausages. The ultimate goal is to discern the role of microbiota in the production of flavoring compounds.

## Materials and methods

### Bacterial strains and culture conditions

Two bacterial strains namely *Sx* YCC3 and *Lp* MSZ2 were used as starter cultures in this study. The bacterial strains were previously isolated from traditional Chinese fermented foods and confirmed by 16S rRNA sequencing. Before inoculation, Mannitol Salt broth and MRS broth were used to culture *Sx* YCC3 and *Lp* MSZ2, respectively, under the standard growth conditions described previously (Chen et al., 2014). Following growth, the bacterial cells were centrifuged at 1000× g for 5 min to produce a pellet, followed by washing two times with 0.85% saline water and storage at 4°C till further use.

### Sausage preparation

Fresh lean pork and back fat (6 months old and healthy black, Shanxi pig) were purchased from the JiaJiaLi market in Taigu, China. Meat was obtained from the same animal and sent to the Meat Laboratory Center of Shanxi Agricultural University of China while maintaining cold chain. The detailed method of preparation for fermented sausage has been described previously (Wang et al., 2022). Briefly, lean pork meat (800 g) was pulverized mechanically through a mincer, mixed with different ingredients (back fat 200 g, table salt 30 g, sugar 50 g, wine 15 ml, sodium erythorbate 0.5 g, and sodium nitrite 0.15 g). The final concentration of starter culture was approximately 10^7^ CFU/g. The contents were mixed thoroughly, and the mixture was stuffed into natural pork casings with a diameter of approximately 3 cm. The sausages were allowed to ferment for 2 days in a humid (90% humidity) environment at 32°C temperature in an incubator (LRHS-150, Yuejin medical apparatus, Shanghai, China). The sausages were fermented for another 12 days at 15°C and 70%–80% relative humidity. The water content of all the sample were <0.86 at ripening. During the ripening process, 10 fermented sausages were randomly selected, of which, 5 sausages were used to assess flavoring compounds, and 5 sausages were stored at −80°C for to assess microbial diversity on days 0, 6, and 12.

### DNA extraction and PCR amplification

Bacterial genomic DNA was extracted from fermented sausages using CTAB (Cetyltrimethylammonium Bromide) method as described previously (Xiao et al., 2021). POCKIT Nucleic Acid Analyzer (Thermo Fisher Scientific; Shanghai, China) was used to evaluate DNA quality and purity. DNA samples were stored at −20°C till further analysis. PCR amplification of the V3-V4 region of the 16S rRNA gene was performed using primer pairs 341F (5′-CCTAYGGGRBGCASCAG-3′) and 806R (5′-GGACTACNNGGGTATCTAAT-3′) (Liu et al., 2014).

### High-throughput sequencing and bioinformatics analysis

Pooled samples of DNA were subject to high throughput sequencing on the Illumina Miseq® platform with 2 × 300 base reads using V3 chemistry as specified by the manufacturer (Illumina, San Diego, California, United States). Data output was demultiplexed with the in-built RTA software. OTUs clustering, species annotation, and alpha diversity analyses were performed as described by Zheng et al. (2018). The OTUs clustering of the effective tags was performed by Uparse software.^1^ The effective tags with more than 97% similarity were assigned to the same OTUs. OTU abundance information was normalized using a standard sequence number corresponding to the sample with the least sequences. QIIME (Version 2.0) was used to calculate alpha and beta diversities based on the normalized data. Chao1 estimator^2^ and the ACE estimator^3^ were used to calculate and ACE, respectively. The mothur software^4^,^5^ was used to calculate Shannon and Simpson’s indices. Mothur^6^ was also used to calculate good coverage.

### Determination of flavor compounds

The presence of flavor compounds in fermented sausages was assessed using headspace solid-phase microextraction-gas chromatography–mass spectrometry (HS-SPME-GC/MS) according to the slightly modified methods of De Lima Alves et al. (2020). Briefly, 5 g of fermented sausage samples were minced and added into a 20 ml headspace vial. Dichlorobenzene was also added to the samples to serve as the internal standard. The flavor compounds Chemical Abstract Service accession numbers were obtained and matched with the corresponding compound in the database.^7^ A flavor compound was confirmed when it share >90% similarity with a particular compound in the database. The following formula was used to assess the concentration of the volatile flavor compounds.


C1=C2×S1/S2

C1 = Relative concentration of the volatile compounds.

S1 = Peak area of the volatile compounds.

S2 = Peak area of the internal standard.

C2 = Concentration of the internal standard.

### Free fatty acid composition

The fatty acid composition was determined using the previously described method by Chen et al. (2017) with slight modifications. Briefly, 15 mg lipid from fermented sausages was added into a tube containing 3 ml of 0.5 M NaOH-methanol and place in a 60°C water bath for 30 min to saponify lipids. The tube was cooled down at room temperature and 2 ml of BF3-MeOH (14%) solution was added followed by further incubation in a water bath at 60°C for 20 min. When cooled, 2 ml of 0.88% NaCl solution and 2 ml of n-hexane were added and the mixture was centrifuged at 4°C and 2000× g for10 min after which, the n-hexane phase was collected and blown with nitrogen gas. The precipitate was re-dissolved in 1 ml n-hexane for further analysis through Gas Chromatography–Mass Spectrometry (GC–MS). GCMS analysis was done with DB-WAX capillary column (30 m × 0.25 mm, 0.25 μm, Agilent, United States; Thermo Fisher Scientific Co., Ltd., China) and using helium with a flow velocity of 1 ml/min as the carrier gas. Both the injection temperature and detection temperature were 250 the split ratio was 10:1. The heating conditions were as follows: The initial temperature was 100°C maintained for 3 min, 10°C/min to 180°C for 3 min, 3°C/min to 250°C for 9 min. Mass spectrometry conditions were as follows: the source temperature was 250°C, the ionization voltage was 70 ev, scan range was from 50 to 550 amu.

### Statistics analysis

All the experiments were performed in triplicate. The numerical data were expressed as mean ± standard deviation. The analysis of variance (ANOVA) was used to assess the differences at different time points using Statistix 9.0 software (United States). A value of *p* < 0.05 was considered significant. Using the SIMCA-P software (Umetrics software V.13.0, Malmö, Sweden), principal component analysis and orthogonal partial least squares discrimination analysis (OPLS-DA) analysis were performed to determine the overall differences between the groups using. To determine the relationship between bacterial abundance, metabolism, and production of volatile compounds, we have used Pearson’s correlation analysis using R package (version 2.15.3, Robert Gentleman, The University of Auckland, New Zealand). The diagrams were obtained by Origin Pro 9.0 software (Origin Lab, Northampton, MA, United States).

## Results

### Taxonomic diversity of the sausage samples during ripening process

Taxonomic diversity of the fermented sausage samples during the ripening process was analyzed by resolving next-generation sequencing reads at the species level and calculating alpha diversity indices (Table 1). The Ace indices reflect the bacterial abundance while Shannon and Simpson’s indices reflect the bacterial diversity in the samples. During the ripening process, the Ace indices showed an increased trend, reaching 69.08 on day 12. Compared to day 0, the bacterial abundance increased significantly throughout the ripening process. Bacterial diversity was positively correlated with Shannon’s index and negatively correlated with Simpson’s index. Overall, Shannon’s index first increases and then decreased during ripening, while the Simpson’s index follows the exact opposite trend.

**Table 1:** Alpha-diversity of bacterial community in fermented sausage during ripening process.

Sample	Valid sequence	Ace	Shannon	Simpson	Coverage/%
YM-0d	29,459	50.76 ± 2.11^c^	1.60 ± 0.03^b^	0.37 ± 0.01^b^	99.99%
YM-6d	31,176	61.16 ± 1.45^b^	1.69 ± 0.01^a^	0.29 ± 0.01^c^	99.99%
YM-12d	41,967	69.08 ± 0.82^a^	1.29 ± 0.09^c^	0.49 ± 0.01^a^	99.99%

Different letters indicate significant differences among groups (*p* < 0.05).

### Relative abundance of bacterial communities in fermented sausages during ripening process

**Figure 1:**
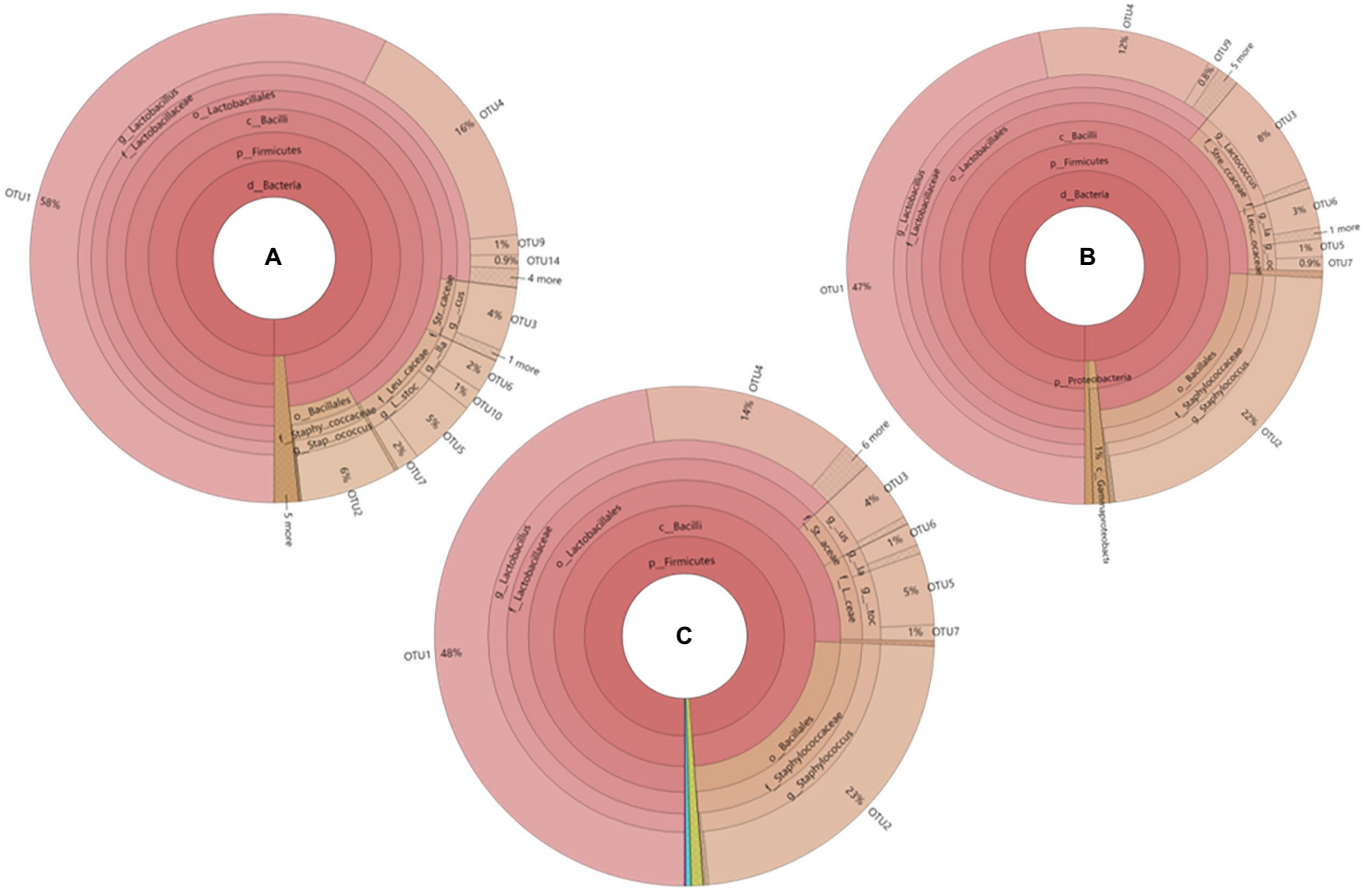
Phylogenetic tree of core OTUs in fermented sausage at different ripening stages **(A)** YM-0d, **(B)** YM-6d, **(C)** YM- 12d.

The phylogenetic tree of core operational taxonomic units (OTUs) in sausages at different ripening stages is shown in Figure 1. A total of 9 core bacterial OTUs were detected in all sausage samples. Among them, OTU1, OTU4, OTU9, and OTU14 were clustered with *Lactobacillus*, and their total relative abundances were 75.9%, 59.8%, and 62.0% on the days 0, 6, and 12 of ripening, respectively. OTU3 was clustered with *Lactococcus*, and the relative abundances were 4.0%, 8.0%, and 4.0% on the days 0, 6, and 12 of ripening, respectively. OTU6 and OTU10 were clustered with *Weissella,* the total relative abundances were 3.0%, 3.0%, and 3.0% on the days 0, 6, and 12 of the ripening process. OTU5 and OTU7 were clustered with *Leuconostoc*, and the total relative abundances were 7.0%, 1.9%, and 6.0%, respectively, on the days 0, 6, and 12 of ripening, OTU2 was clustered with *Staphylococcus*, and the relative abundances were 6.0%, 22.0%, and 23.0% on the days 0, 6, and 12 of ripening, correspondingly.

**Figure 2:**
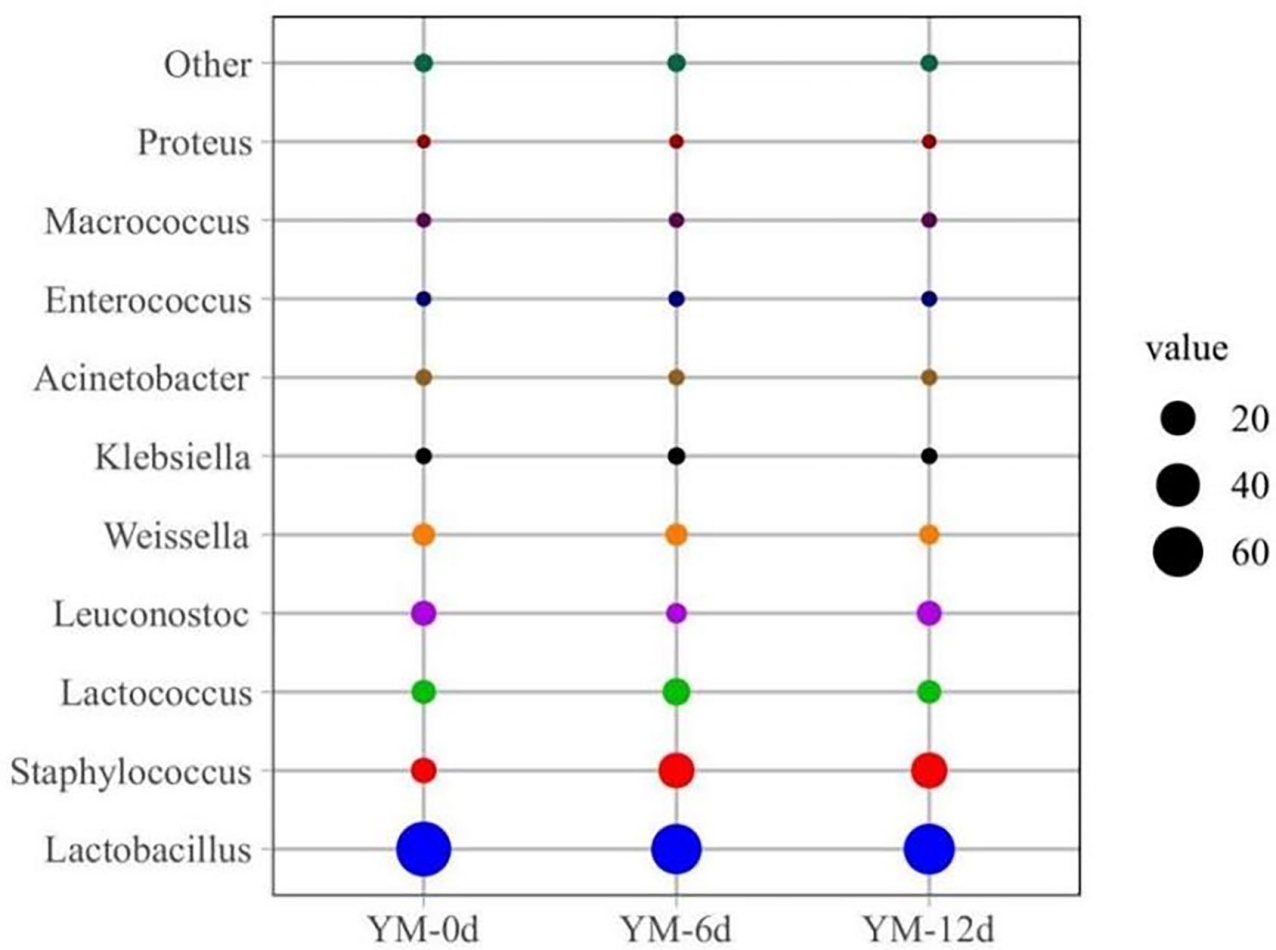
Relative abundance of bacteria community at genus levels in fermented sausage during ripening process.

The relative abundance of bacteria at the genus level in different ripening stages of fermented sausages was also assessed (Figure 2). *Lactobacillus*, *Staphylococcus*, *Lactococcus*, *Leuconostoc,* and *Weissella* were the dominant bacterial genera found in all fermented sausage samples during the ripening process. Overall, on day 0, the relative abundances of *Lactobacillus* and *Staphylococcus* were 76.96% and 6.44%, respectively. On the 12th day of ripening, the relative abundance of *Lactobacillus* decreased to 63.03%, while that of *Staphylococcus* increased to 22.96%. As shown in Figure 2, other genera showed similar upward and downward trends in relative abundance.

**Figure 3:**
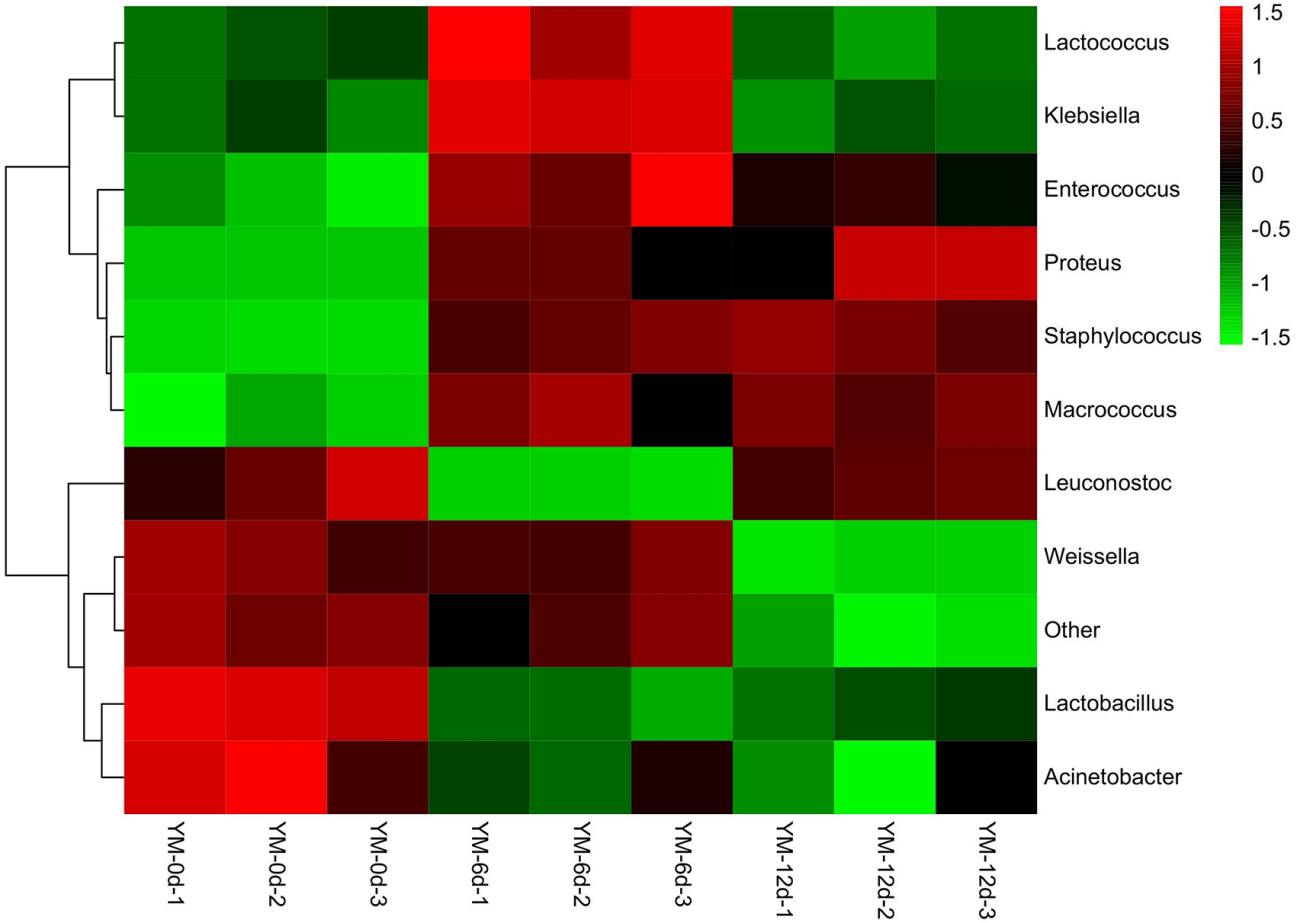
Heat map of bacterial community abundance in fermented sausage at genus level during ripening process.

The relative abundance of bacteria at the genus level in the samples was plotted into a cluster heat map to discern the changes more clearly in bacterial abundance of fermented sausages during ripening (Figure 3). There were significant differences in the relative abundance of bacteria in sausages at different ripening stages.

### Predicted functional analysis of bacterial microbiome during the ripening process in fermented sausages

**Figure 4:**
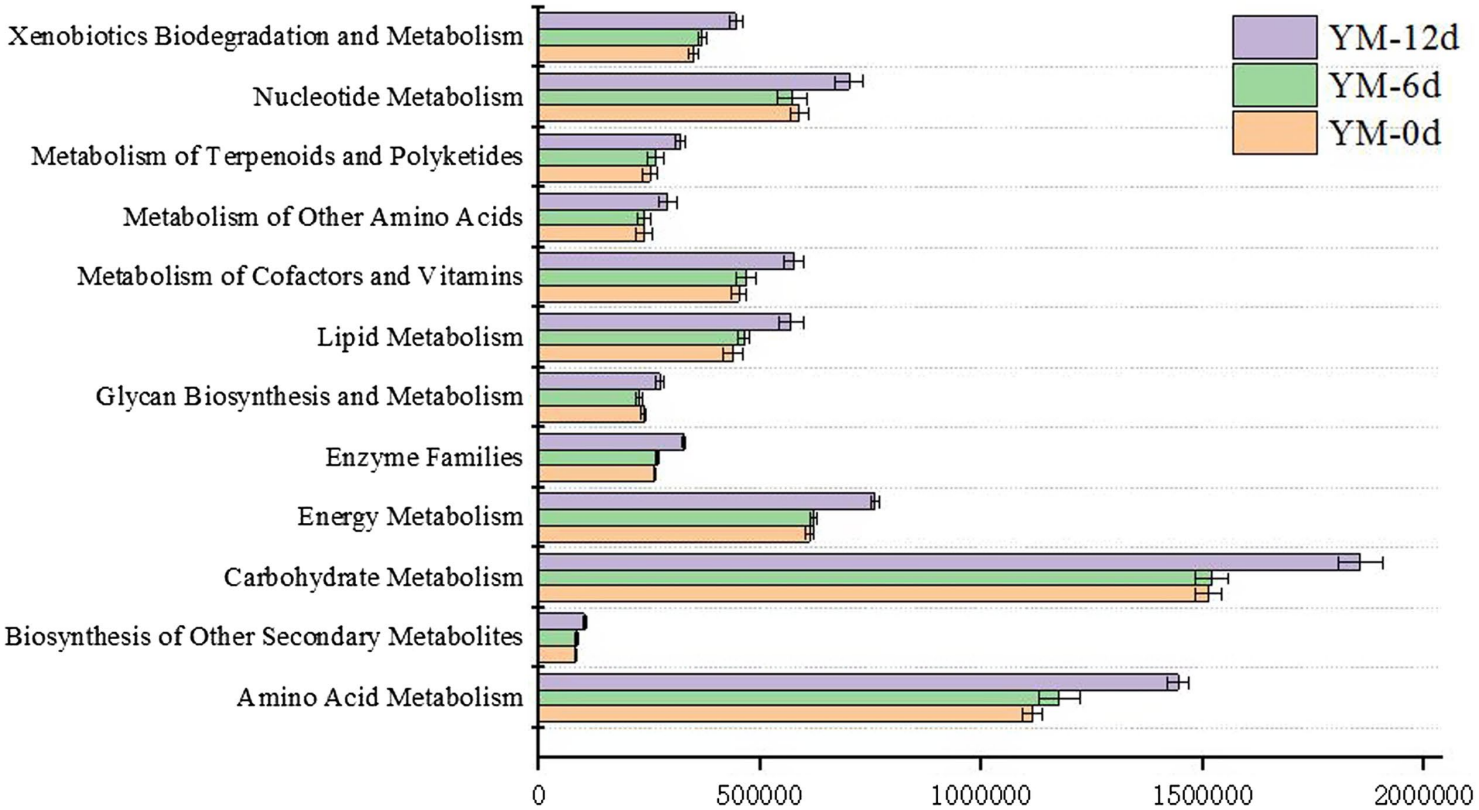
Prediction of bacterial metabolic function in fermented sausage.

To further reveal bacterial metabolic pathways and functional potential, KEGG functional orthologs were inferred by the PICRUSt algorithm for 16S-based bacterial members. Figure 4 displayed several metabolic predictive functional pathways in fermented sausages during the ripening process, which all showed an increasing trend as the ripening process proceeded. Among the several metabolic pathways, the main metabolic pathways were amino acid metabolism (Supplementary Figure S1), nucleotide metabolism (Supplementary Figure S2), lipid metabolism (Supplementary Figure S3), and carbohydrate metabolism (Supplementary Figure S4). The abundances of these four metabolic pathways on the 12th day of ripening were e 1,856,164, 701,389, 570,705, and 1,444,202, respectively, which were significantly higher than that on the 0th day of ripening.

### Profile of volatile compounds in fermented sausages at different ripening stages

The composition and content of volatile flavor compounds in fermented sausages at different ripening periods were presented in Table 2. A total of 63 volatile compounds were detected during the ripening process, including 25 aldehydes, 8 ketones, 12 alcohols, 10 esters, and 8 acids. 26, 37, and 43 flavor compounds were detected on the days 0, 6, and 12 of sausage ripening, respectively, indicating that ripening time plays an important role in the formation of sausage flavor. Aldehyde compounds have a lower flavor threshold and are vital flavor compounds in fermented sausages. On day 0 of ripening, there were 15 kinds of aldehydes in the fermented sausage, while on day 12 of ripening, there were 22 kinds of aldehydes in the sausage, and the content was significantly higher than that on day 0. Hexanal, heptaldehyde, and nonanal are representative aldehydes in fermented sausages, which can impart grassy, greasy, and floral aromas to sausages (Hu et al., 2020b). On day 6 of ripening, the content of heptaldehyde reached a maximum of 333.97 μg/kg, and on day 12 of ripening, the contents of hexanal and nonanal reached the highest value of 207.76 and 2828.93 μg/kg, respectively. E,E-2,4-decadienal, lily aldehyde, trans-2,4-heptadienal, pentanal, and other compounds were detected during the ripening process, and these compounds can endow sausage lily aroma, creamy aroma, and other smells.

**Table 2:** Changes of volatile flavor compounds during the ripening of fermented sausages.

Volatile compound	Retention time (RT)	YM-0d	YM-6d	YM-12d
Hexanal (A1)	5.72	81.44 ± 10.34^C^	162.80 ± 19.10^B^	207.76 ± 6.17^A^
trans-2-Hexenal (A2)	7.42	60.51 ± 9.53^C^	96.22 ± 8.05^A^	74.33 ± 5.04^B^
Heptaldehyde (A3)	9.13	156.61 ± 10.01^C^	333.97 ± 6.65^A^	259.69 ± 17.02^B^
2-Heptenal, (2Z)–(A4)	11.22	332.85 ± 23.06^C^	1625.51 ± 48.8^A^	1463.59 ± 29.35^B^
(E)-2-Octenal (A5)	14.69	163.57 ± 12.36^C^	590.62 ± 14.93^B^	637.82 ± 20.71^A^
1-Nonanal (A6)	16.92	1435.4 ± 200.45^C^	1782.22 ± 71.53^B^	2311.95 ± 40.11^A^
2-Nonenal (A7)	19.00	156.37 ± 12.14^B^	506.80 ± 35.29^A^	516.98 ± 10.36^A^
(Z)-4-Decenal (A8)	20.37	n.d.	n.d.	71.90 ± 1.01^A^
2-Decenal (A9)	22.66	457.78 ± 19.31^C^	1427.90 ± 12.58^B^	2249.98 ± 40.66^A^
2-Undecenal (A10)	26.14	365.19 ± 19.11^C^	1515.55 ± 77.15^B^	1900.83 ± 48.42^A^
Benzylcarboxaldehyde (A11)	14.59	n.d.	27.13 ± 2.75^A^	n.d.
Octanal (A12)	13.02	334.72 ± 13.86^B^	503.46 ± 48.95^A^	506.59 ± 17.33^A^
Decanal (A13)	20.69	35.07 ± 5.53^B^	70.63 ± 1.62^A^	75.10 ± 3.28^A^
2,4-Nonadienal (A14)	21.05	24.09 ± 1.96^B^	n.d.	115.13 ± 2.36^A^
(E,E)-2,4-Decadienal (A15)	23.79	n.d.	381.87 ± 6.70^B^	935.40 ± 12.56^A^
Undecanal (A16)	24.24	16.58 ± 3.34^C^	60.91 ± 4.07^A^	52.66 ± 2.34^B^
Dodecyl aldehyde (A17)	27.59	23.81 ± 2.44^C^	217.78 ± 12.12^A^	128.60 ± 1.88^B^
Lily aldehyde (A18)	31.22	n.d.	39.70 ± 3.18^B^	49.39 ± 3.15^A^
Tetradecanal (A19)	39.39	31.37 ± 1.46^C^	221.04 ± 6.62^B^	375.24 ± 5.84^A^
cis-4-Heptenal (A20)	8.95	n.d.	21.28 ± 1.33^A^	n.d.
2,4-Decadienal (A21)	23.90	n.d.	n.d.	54.88 ± 1.28^A^
Pentanal (A22)	3.41	n.d.	155.07 ± 5.25^B^	357.70 ± 6.32^A^
trans-2,4-Heptadienal (A23)	13.36	n.d.	89.32 ± 4.72^A^	n.d.
5-Octadecenal (A24)	33.41	n.d.	64.40 ± 3.65^A^	n.d.
cis-9-Tetradecenal (A25)	30.88	n.d.	n.d.	49.54 ± 1.64^A^
Nerylacetone (A26)	2,878	n.d.	n.d.	49.54 ± 2.36^A^
2-Heptanone (A27)	8.69	16.60 ± 2.71^B^	n.d.	31.91 ± 1.34^A^
5-Decanone (A28)	22.06	n.d.	95.43 ± 6.07^A^	n.d.
Geranylacetone (A29)	28.76	n.d.	106.40 ± 7.89^A^	n.d.
1-Octen-3-one (A30)	12.00	n.d.	41.37 ± 3.29^B^	50.14 ± 1.16^A^
2-Undecanone (A31)	23.70	n.d.	69.35 ± 5.79^A^	55.76 ± 1.27^B^
2-Tridecanone (A32)	30.25	n.d.	n.d.	117.02 ± 2.10^B^
2-Dodecanone (A33)	30.30	9.54 ± 0.65^A^	n.d.	n.d.
1-Hexanol (A34)	8.07	n.d.	n.d.	75.09 ± 1.79^A^
1-Octen-3-ol (A35)	12.17	144.73 ± 10.75^C^	597.72 ± 29.81^B^	719.48 ± 20.39^A^
2-Pentadecyn-1-ol (A36)	25.62	n.d.	n.d.	78.11 ± 0.92^A^
1-Pentanol (A37)	4.91	n.d.	85.61 ± 7.21^B^	110.41 ± 3.16^A^
n-Heptanol (A38)	11.84	13.03 ± 1.17^B^	n.d.	43.72 ± 1.58^A^
2,4-Dimethylpent-1-en-3-ol (A39)	16.55	n.d.	n.d.	407.25 ± 10.47A
Phenylethyl Alcohol (A40)	17.39	n.d.	86.03 ± 6.96^A^	32.78 ± 2.96^B^
2,4-Decadien-1-ol (A41)	20.36	19.85 ± 2.86^A^	n.d.	n.d.
2-Hexadecanol (A42)	23.38	n.d.	79.16 ± 6.86^A^	n.d.
2-Octyldecanol (A43)	32.11	n.d.	n.d.	77.23 ± 1.06
Ethanol (A44)	1.83	456.84 ± 27.71^A^	n.d.	n.d.
Isopinocarveol (A45)	18.50	12.80 ± 2.29^B^	34.31 ± 2.25^A^	n.d.
Propionic acid, 2-methyl-, 1-(1,1-dimethylethyl)-2-methyl-1,3-propanediyl ester (A46)	33.18	16.25 ± 3.03^B^	n.d.	72.95 ± 1.85^A^
3,6-Octadecadiynoic acid, methyl ester (A47)	13.40	10.19 ± 1.00^A^	n.d.	n.d.
Cyclopropanetetradecanoic acid, 2-octyl-, methyl ester (A48)	28.32	n.d.	26.02 ± 2.41^A^	n.d.
4-Hydroxynonanoic acid gamma-lactone (A49)	25.95	n.d.	n.d.	129.20 ± 1.97^A^
(E)-9-Tetradecen-1-olacetate (A50)	24.99	n.d.	n.d.	56.13 ± 1.05^A^
Ethyl caprate (A51)	27.11	n.d.	49.28 ± 3.03^A^	35.18 ± 1.11^B^
Crotonic acid, menthyl ester (A52)	29.66	n.d.	n.d.	63.32 ± 2.76^A^
Hexanoic acid, 2-phenylethyl ester (A53)	8.68	n.d.	39.04 ± 1.82^A^	n.d.
Ethyl cis-4-decenoate (A54)	20.35	n.d.	119.96 ± 10.26^A^	n.d.
12,15-Octadecadiynoic acid, methyl ester (A55)	32.35	n.d.	23.26 ± 0.93^A^	n.d.
trans-2-Undecenoic acid (A56)	27.12	n.d.	35.23 ± 3.55^A^	n.d.
Nonanoic acid (A57)	23.29	n.d.	n.d.	242.59 ± 9.46^A^
7-Nonynoic acid (A58)	12.82	n.d.	n.d.	72.87 ± 1.94^A^
Malonamic acid (A59)	1.96	157.04 ± 17.60^A^	n.d.	n.d.
Octanoic Acid (A60)	19.81	n.d.	n.d.	55.81 ± 0.99^A^
n-Decanoic acid (61)	23.36	n.d.	n.d.	109.79 ± 2.37^A^
Pterin-6-carboxylic acid (A61)	6.30	13.59 ± 1.60^A^	n.d.	n.d.
Isovaleric acid (A62)	7.28	n.d.	28.82 ± 2.32^A^	n.d.

n.d.: volatile flavor compounds not detected. a–d: different letters in the same row indicate significant differences (*p* < 0.05). A1–A762 following the name of volatile compound represents the number of flavor substances. RT: retention time (min).

Ketones are typical flavor compounds in fermented sausages, which can impart floral, creamy, fatty, and fruity flavors to fermented sausages (Zhang et al., 2019). 2, 4, and 3 ketones were detected on days 0, 6, and 12 of sausage ripening, and the types and contents of ketones detected on days 6 and 12 were pointedly higher than that on day 0, indicating that ketones are mainly formed during the ripening process, which may have a potential effect on improving sausage flavor.

Acids are formed through carbohydrates metabolized by bacteria and are the most characteristic flavor compounds in sausages. They are also precursors of other flavor compounds (Xiao et al., 2021). In this experiment, two kinds of acids were detected on day 0 of ripening, and four kinds of acids were detected on day 12 of ripening, namely nonanoic acid, 7-nonenoic acid, octanoic acid, and n-decanoic acid. The total amount of acids on day 12 of ripening (481.06 ug/kg) was significantly higher than that on day 0 (170.63 ug/kg), indicating that a large number of acids were produced during the ripening process.

Compared with aldehydes and ketones, alcohols have a higher flavor threshold and have little direct effect on the flavor of sausages, but they are precursors of other flavors, so they also play an important role in the formation of sausage flavors (Toldra, 1998). 5, 5, and 8 alcohols were detected on days 0, 6, and 12 of sausage ripening, respectively. Phenylethyl alcohol, 1-octen-3-ol, and 1-pentanol are alcohols that contribute more to flavor. 1-octen-3-ol is an important alcohol substance in fermented sausage, also known as mushroom alcohol, which can impart mushroom flavor to sausage (Wang Z. et al., 2020), and its content increases significantly during the ripening process. 1-pentanol can give sausage oil flavor, detected on days 6 and 12 of ripening, and the content on day 12 of ripening was 113.64 μg/kg, which was significantly higher than that on day 6 of ripening. Phenylethyl alcohol is an aromatic alcohol, which can impart a rose aroma to sausages, and its content was highest (86.03 μg/kg) on day 6. Other alcohols play an important role in the formation of esters. Esters have lower flavor thresholds and can impart fruity and floral aromas to sausages. 2, 5, and 5 kinds of esters were detected on days 0, 6, and 12 of sausage ripening, indicating that esters were mainly formed during the ripening process.

To sum up, the flavor compounds in sausages are mainly formed during the ripening process, and as the ripening time increases, the types and contents of flavor compounds show a momentous upward trend. Wang Z. et al. (2020) found that the flavor compounds of sour fish were mainly formed during the processing.

**Figure 5:**
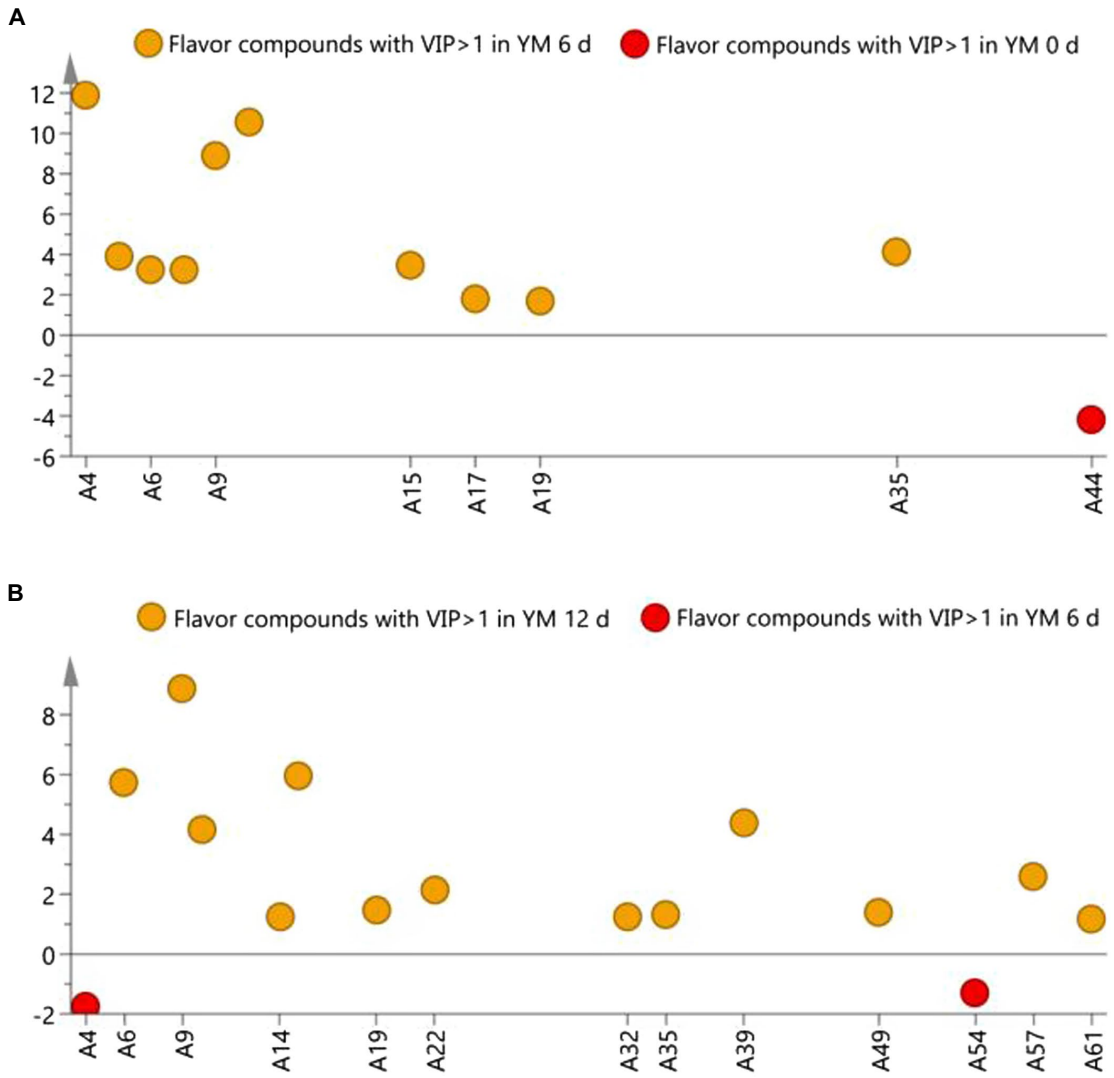
S-line scatter plot based on OPLS-DA model analysis.

Compounds with a VIP ≥ 1 is generally identified as differential flavor compounds (da Silva et al., 2017). It can be seen from (Figure 5) that the differential flavor compounds on day 0 and day 6 of ripening are 2-heptenal, (2Z)–(A4), 2-undecenal (A10), 2-decenal (A9), ethanol (A44), E-2-octenal (A5), E,E-2,4-decadienal (A15), 1-nonanal (A6), 2-nonanal (A7), dodecyl aldehyde (A17), tetradecanal (A19), 1-octen-3-ol (A35). The different flavor compounds on day 6 and day 12 of ripening are 2-decenal (A9), 2-heptenal,(2Z)–(A4), (E,E)-2,4-decadienal (A15), 1-nonanal (A6), 2,4-dimethylpent-1-en-3-ol (A39), 2-undecenal (A10), pentanal (A22), nonanoic acid (A57), 1-octen-3-ol (A35), tetradecanal (A19), 4-hydroxynonanoic acid gamma-lactone (A49), ethyl cis-4-decenoate (A54), 2-tridecanone (A32), 2,4-nonadienal (A14), pterin-6-carboxylic acid (A61). 2-heptenal, (2Z)–(A4),2-decenal (A9), 2-undecenal (A10), 1-nonanal (A6), E, E-2,4-decadienal (A15), tetradecanal (A19), 1-octen-3-ol (A35) are common differential flavor compounds, whose content on days 6, 12 were significantly higher than that on day 0.

### Changes of free fatty acid components during maturation of fermented sausages

Table 3 displays the free fatty acid (FFA) composition of fermented sausage during the ripening process. A total of 10 saturated fatty acids (SFA), 6 monounsaturated fatty acids (MUFA), and 7 polyunsaturated fatty acids (PUFA) were detected in all sausage samples. The contents of each free fatty acid increased significantly from the 6th to the 12th day of ripening. In general, MUFA > SFA > PUFA at day12 of ripening. The total amounts of SFA, MUFA, and PUFA were 9603.98, 11940.60, and 2040.72 mg/kg on day 12 of ripening, respectively, which were significantly higher than those on day 0 of ripening (*p* < 0.05). Palmitic acid (C16:0) and stearic acid (C18:0) were the primary SFA observed in sausages, and their contents were 5040.95 and 3483.34 mg/kg at 12 days of ripening, respectively. Oleic acid (C18:1) was the major MUFA found in sausages. Initially, its concentration was 941.83 mg/kg, rising to 11552.98 mg/kg over the 12-day ripening. Among PUFA, the highest content was linoleic acid with 1777.62 mg/kg at day 12 of ripening, followed by arachidonic acid (C20:2), arachidonic acid (C20:4), linolenic acid (C18:3), and docosahexaenoic acid (DHA, C22:6) with 126.75 mg/kg, 50.19 mg/kg, 50.19 mg/kg, 50.19 mg/kg, and 50.19 mg/kg, respectively, at day 12 of ripening, which were significantly higher (*p* < 0.05) than that at the day 0 of ripening.

**Table 3:** Changes of free fatty acid components during maturation of fermented sausages (mg/kg).

	YM-0d	YM-6d	YM-12d
C4:0	107.49 ± 28.88^c^	129.14 ± 11.20^b^	261.93 ± 18.39^a^
C6:0	1.64 ± 0.26^c^	2.39 ± 0.42^b^	7.73 ± 0.29^a^
C10:0	1.18 ± 0.02^c^	3.00 ± 0.42^b^	11.67 ± 0.10^a^
C12:0	1.17 ± 0.19^c^	3.00 ± 0.33^b^	11.11 ± 0.33^a^
C14:0	31.57 ± 2.76^c^	93.25 ± 10.55^b^	392.17 ± 3.58^a^
C15:0	0.81 ± 0.14^c^	1.39 ± 0.18^b^	5.24 ± 0.23^a^
C16:0	402.19 ± 40.68^c^	1146.26 ± 118.17^b^	5040.95 ± 36.80^a^
C17:0	2.23 ± 0.37^c^	5.90 ± 0.50^b^	24.83 ± 0.20^a^
C18:0	277.71 ± 25.81^c^	742.00 ± 67.75^b^	3483.34 ± 28.92^a^
C20:0	29.42 ± 2.45^c^	86.21 ± 7.30^b^	376.68 ± 6.55^a^
SFA	854.22 ± 98.85^c^	2209.53 ± 193.15^b^	9603.98 ± 57.05^a^
C14:1	0.52 ± 0.17^b^	0.65 ± 0.01^b^	2.08 ± 0.25^a^
C16:1	23.71 ± 2.09^c^	71.06 ± 7.63^b^	289.81 ± 1.56^a^
C17:1	1.89 ± 0.29^c^	4.80 ± 0.38^b^	20.07 ± 0.37^a^
C18:1	941.83 ± 102.84^c^	2745.44 ± 267.21^b^	11552.98 ± 119.11^a^
C20:1	5.54 ± 0.07^c^	14.89 ± 1.31^b^	59.90 ± 0.54^a^
C22:1	1.62 ± 0.08^c^	3.91 ± 0.33^b^	15.76 ± 1.02^a^
MUFA	975.12 ± 105.07^c^	2840.75 ± 276.88^b^	11940.60 ± 121.43^a^
C18:2	154.99 ± 12.34^c^	433.21 ± 39.98^b^	1777.65 ± 17.35^a^
C20:2	10.50 ± 0.96^c^	29.66 ± 2.51^b^	126.75 ± 2.23^a^
C18:3	2.99 ± 0.40^c^	7.95 ± 0.67^b^	38.02 ± 0.30^a^
C20:3	0.55 ± 0.27^c^	1.03 ± 0.03^b^	3.37 ± 0.07^a^
C20:3	1.58 ± 0.30^c^	4.55 ± 0.11^b^	17.09 ± 0.48^a^
C20:4	4.78 ± 0.48^c^	12.18 ± 0.97^b^	50.19 ± 0.93^a^
C22:6	2.82 ± 0.76^c^	6.55 ± 0.71^b^	27.64 ± 1.92^a^
PUFA	178.21 ± 14.29^c^	495.14 ± 44.98^b^	2040.72 ± 21.52^a^
total	2008.73 ± 217.83^c^	5548.42 ± 515.43^b^	23596.97 ± 190.61^a^

Different large letters indicate significant differences among groups (*p* < 0.05). YM-0d, YM-6d and YM-12d, represents the groups at day 0, 6^th^ and 12^th^.

### Correlation between microbial flora, functional metabolic abundance, and characteristic flavor compounds

**Figure 6:**
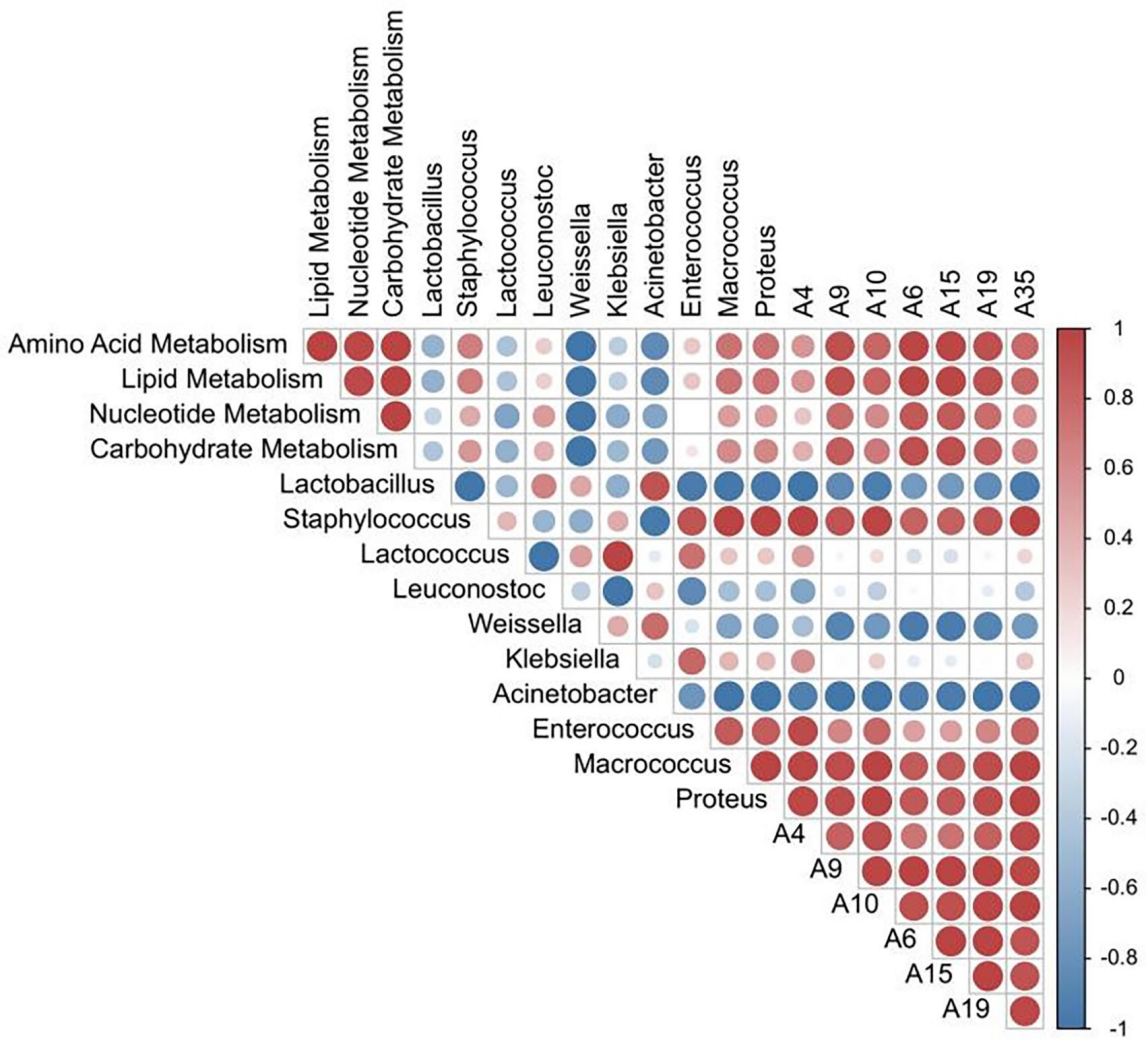
Heat map of the correlation between the relative abundance of dominant bacteria, functional metabolic abundance, and differential flavor compounds.

**Figure 7:**
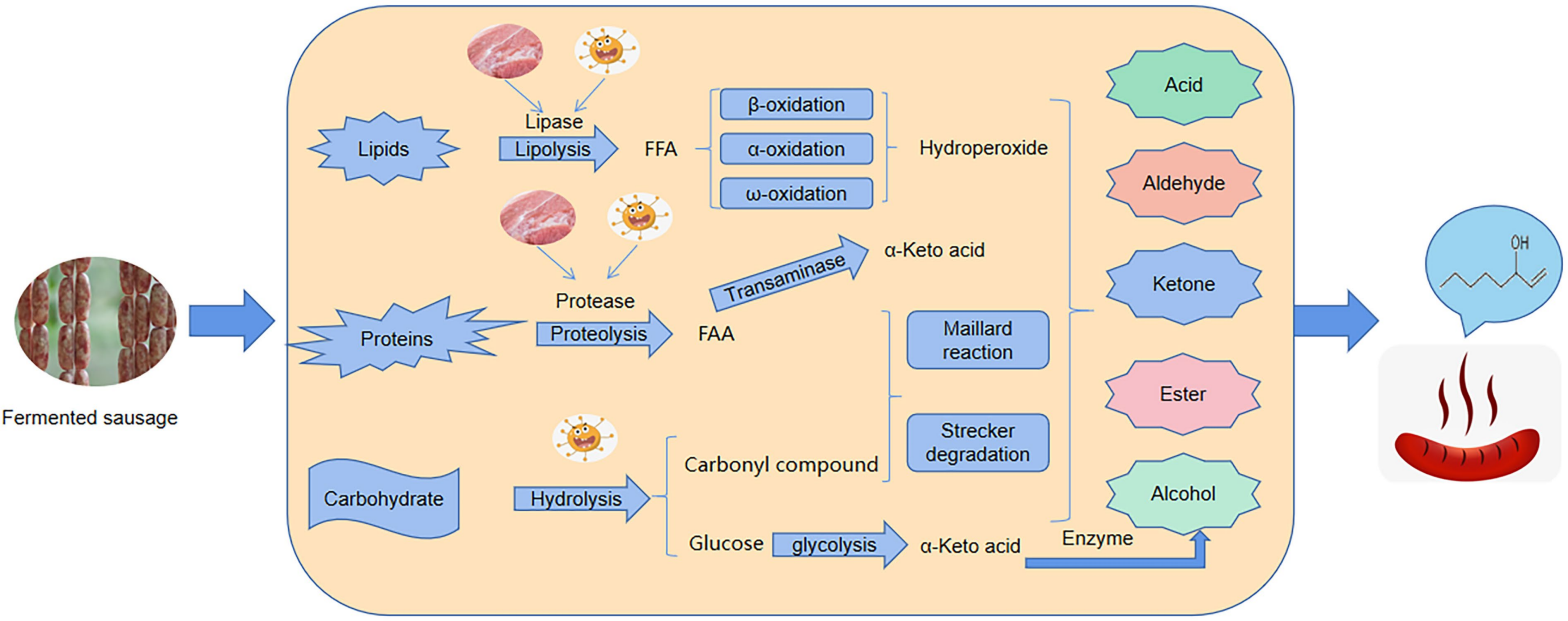
The schematic diagram of mechanism of microorganisms on the formation of flavor substances in fermented sausages.

Correlation analysis between the relative abundance of dominant bacterial flora, functional metabolism, and the content of differential flavor compounds was also performed. As shown in Figure 6, the relative abundance of *Staphylococcus* and *Leuconostoc* are positively correlated with carbohydrate, nucleotide, lipid metabolism, and amino acid metabolism. The correlation coefficient between *Staphylococcus* and lipid and amino acid metabolism was 0.67 and 0.68, respectively. Also, the abundance of *Lactobacillus* is negatively correlated with lipid metabolism. *Staphylococcus* was also positively correlated with the contents of 7 differential flavor compounds, and the correlation coefficient was 0.82–1.00, indicating that *Staphylococcus* played an imperative role in the formation of sausage flavor during the ripening process. Similarly, the common metabolic pathways were also positively correlated with differential flavor compounds. For example, the correlation coefficients between 2-decenal and carbohydrate metabolism, nucleotide metabolism, lipid metabolism, and amino acid metabolism were 0.85, 0.78, 0.93, and 0.92, respectively. The correlation coefficients between 1-octen-3-ol (A35) and carbohydrate metabolism, nucleotide metabolism, lipid metabolism, and amino acid metabolism were 0.68, 0.58, 0.79, and 0.78, respectively. The schematic diagram of mechanism of microorganisms on the formation of flavor substances in fermented sausages has been shown in Figure 7.

## Discussion

Food fermentation is recognized as one of the most effective and economical methods of food preservation and processing in human civilization. The process is driven by the microbiome, in which the microbiota plays different and important functions, including but not limited to the production of flavor compounds, inhibition of the growth and reproduction of exogenous and pathogenic bacteria, reduction biogenic amines and nitrite contents, and improvement of the safety and quality of the final product (Wang Z. et al., 2021). In the current study, we have reported bacterial microbiome diversity during the ripening of Chinese fermented sausages and its impact on flavor compounds. Results show that bacterial diversity decreases as the fermentation process proceeds. This could be explained by the fact that the internal environment of the sausage was suitable for the growth of several microorganisms during the early stages of fermentation. At the later stage of ripening, acid accumulation and pH reduction may inhibit the growth of microorganisms with poor acid tolerance. Another possible reason may be that LAB and *Staphylococcus* inhibit the growth of other bacteria as the fermentation process advances. Similar findings have also been reported elsewhere (Xiao et al., 2021).

The relative abundance of different bacterial genera also varied greatly during fermentation, which affected the quality and safety of fermented sausages. Our study showed that *Lactobacillus* and *Staphylococcus* became the dominant bacteria during ripening. Similar findings have also been reported previously by Papamanoli et al. (2002), who found that *Lactobacillus* and *Staphylococcus* were the dominant bacteria in Cantonese sausages. However, the relative abundance of other microorganisms decreased during ripening, which may be attributed to the inhibitory effects of bacteriocin produced by *Lactobacillus*. Microbial growth inhibition by LAB strains has been also reported in other studies (Yin et al., 2002; Wang J. et al., 2020). The fermented sausages are also easily contaminated by different microorganisms in the environment during processing, resulting in the deterioration of product. *Enterococcus*, *Klebsiella*, *Acinetobacter,* and *Proteus* are common spoilage bacteria in fermented sausages (Wang et al., 2018). In this context, the role of LAB species is also crucial because of its ability to rapidly produce acid, which can quickly reduce the pH value of sausage and effectively inhibit the growth of spoilage and pathogenic bacteria. Furthermore, the antioxidant effect of LAB inhibits the rancidity of sausage (Feng et al., 2021). We have also observed an increase in the relative abundance of *Staphylococcus* during the ripening of fermented sausages. These findings are in concordance with the previous reports by Rodríguez et al. (1994) and Lorenzo et al. (2012) who found an increase in *Staphylococcus* abundance during fermentation and processing of Italian and Spanish ham. The consequence is beneficial since research evidence suggests that *Staphylococcus* has nitrite reductase activity that helps to improve the color of fermented sausages. *Staphylococcu*s also hydrolyze lipids and proteins to generate a large amount of free fatty acids (especially unsaturated fatty acids) and free amino acids, which are further degraded and oxidized to generate acids, aldehydes, ketones, and esters (Xiao et al., 2021). At the same time, the relatively active esterase activity of *Staphylococcu*s generates many esters that greatly improve and enhance the flavor of fermented sausages (Feng et al., 2021).

To examine the role of microorganisms during ripening, PICRUSt is used to explore the KEGG ortholog count of functional genes from microbial communities. Functional analysis reveals several important metabolic pathways, especially carbohydrate metabolism, lipid metabolism, and amino acid metabolism, which were involved in flavor formation during the fermentation of sausages (Figure 7). The highest abundance was observed in the carbohydrate metabolism pathway at different stages of ripening, so the carbohydrate metabolism of bacteria in sausage plays a crucial role in the ripening process. On the one hand, it provides energy for the metabolic activities of the microbes themselves (Zhao et al., 2021). On the other hand, bacterial carbohydrate metabolism can also produce a large number of organic acid products which can give sausages a unique sour taste and reduce the pH of sausages, thereby ensuring their safety of sausages. In addition, some key flavor compounds in sausages are formed by carbohydrate metabolism (Chen et al., 2014). Lipolysis and proteolysis are considered to be important processes for developing flavor in fermented sausages (Xiao et al., 2021). Similarly, our results also showed that lipid metabolism is also the main pathway for the formation of flavor compounds (Feng et al., 2021). During the ripening process, triglycerides and glycerophospholipids are hydrolyzed under the action of endogenous lipases to generate many flavor precursors (free fatty acids). In our study, glycerophospholipid metabolic pathways are highly abundant and are an important source of polyunsaturated fatty acids (Huang et al., 2014). Apart from endogenous lipases, research reports that *Staphylococcus* can also secrete lipase driving the hydrolysis of lipids to generate a large amount of free fatty acids (Feng et al., 2021). The free fatty acids are further oxidized to peroxides followed by a complex process of decomposition to form flavor compounds or flavor precursors. There exist several different pathways of fatty acid catabolism, the most important of which is β-oxidation, which produces the ketones under the action of *Staphylococcus* (Hu et al., 2020a). Lipid oxidation is further divided into enzymatic oxidation and auto-oxidation. Enzymatic oxidation occurs mainly by the action of lipoxygenase. Auto-oxidation breakdown unsaturated fatty acids to generate hydroperoxides that are further decomposed to form flavor compounds (Huang et al., 2014). For example, hexanal, heptanal, nonanal, 1-octen-3-ol, and 1-pentanol are generated by auto-oxidation of linoleic acid and arachidonic acid (Muriel et al., 2004). In the current study, we found an increase in the content of lipid metabolites such as hexanal, heptanal, nonanal, 1-octen-3-ol, and 1-pentanol as the ripening advanced. The increase indicates that the lipid metabolism pathway plays a crucial role in flavor formation in the ripening process. Furthermore, hydrolysis of proteins by the action of proteases produces many amino acids and oligopeptides, which are important flavor precursor compounds (Benito et al., 2005). In this study, the amino acid metabolic pathway with the highest abundance in amino acid-related enzymes was observed during the ripening of the sausages. Previously, several studies have reported that aromatic compounds are formed by the catabolism of amino acids (Leucine, phenylalanine, isoleucine, proline, arginine, etc) during fermentation and the bacterial flora such as *Staphylococcus* involve in flavor production (Ganesan et al., 2004; Hozbo et al., 2006; Hou et al., 2013; Dumitriu et al., 2019). The high abundance of all these different metabolic pathways gradually increased during the ripening process and so the content and types of flavor compounds in sausages on day 12 of ripening were significantly higher than that on day 0 of ripening, some compounds like hexanal, heptanal, nonanal, and 1-octen-3-ol were identified as the characteristic flavor compounds in fermented sausages. These compounds give the fermented sausages; the lemon, green grass, citrus, fruit, mushroom, flower, tangerine, and other odors and thus play an important role in improving the flavor of the fermented sausage (Wang et al., 2022). The correlation analysis shows that *Lactobacillus plantarum* MSZ2 strain used as starter culture in this study did not possess lipolysis ability and hence does not contribute to the formation of flavor compounds, as opposed to previous reports (Thierry et al., 2015). In contrast, *Staphylococcus*, the second most dominant bacterial genera significantly connected with carbohydrate metabolism, nucleotide metabolism, lipid metabolism, and amino acid metabolism and exhibited a high positive correlation with seven different flavor compounds, thus confirming previous studies by Zheng et al. (2018). The underlying mechanism may be attributed to the fact that the oxidation and hydrolysis of amino acids and lipids result in flavor formation and enhancement (Jeong et al., 2014).

## Conclusion

In conclusion, *Lactobacillus plantarum* MSZ2 and *Staphylococcus xylosus* YCC3 were used as mixed starter cultures to produce fermented sausages for analyzing the correlation between bacterial diversity, flavor compounds, and metabolic pathways. Overall, the bacterial diversity of fermented sausages increased at first and then decreased during the ripening process, while the bacterial richness increased with time. Through KEGG functional prediction, it was found that the metabolic abundance of bacteria in sausage increased during the ripening process, and the main metabolic pathways included carbohydrate metabolism, nucleotide metabolism, lipid metabolism, and amino acid metabolism. Through correlation analysis, it was observed that *Staphylococcus* and *Leuconostoc* can promote the abundance of carbohydrate metabolism, nucleotide metabolism, lipid metabolism, and amino acid metabolism, and then promote the formation of characteristic flavor compounds of fermented sausage. Therefore, the ripening process plays an important role in improving the bacterial quality of fermented sausages and promoting the accumulation of flavor compounds in sausages. Future studies should focus on a more comprehensive analysis of bacterial species, environmental conditions, and their impact on flavor compounds production in Chinese sausages.

## Data availability statement

The date presented in the study are included in the article/supplementary material, further inquiries can be directed to the corresponding author.

## Author contributions

JW: investigation, data curation, formal analysis, and writing—review and editing. TA: data curation, formal analysis, and writing—original draft. RB: methodology, software, and writing—original draft. XZ: writing—review and editing and revision. MS: writing and revision. MYS: writing—review and editing. AK: methodology and software. AD: writing-review and editing and revision. YZ: funding acquisition, resources, supervision, and project administration. All authors contributed to the article and approved the submitted version.

## Funding

This research was funded by the Natural Science Research Projects of Shanxi Province (20210302123400) in China and the Key R&D Program (Agricultural Field) of Shanxi Province (201903D211008).

## Conflict of interest

The authors declare that the research was conducted in the absence of any commercial or financial relationships that could be construed as a potential conflict of interest.

## Publisher’s note

All claims expressed in this article are solely those of the authors and do not necessarily represent those of their affiliated organizations, or those of the publisher, the editors and the reviewers. Any product that may be evaluated in this article, or claim that may be made by its manufacturer, is not guaranteed or endorsed by the publisher.

## Footnotes

^1^ http://drive5.com/uparse/

^2^ http://www.mothur.org/wiki/Chao

^3^ http://www.mothur.org/wiki/Ace

^4^ http://www.mothur.org/wiki/Shannon

^5^ http://www.mothur.org/wiki/Simpson

^6^ http://www.mothur.org/wiki/Coverage

^7^ http://www.flavornet.org/flavornet.html
