# Supplementary material for: Dynamic change of bacterial diversity, metabolic pathways, and flavor during ripening of the Chinese fermented sausage
Source: Front Microbiol. 2022 Oct 4;13:990606. doi: 10.3389/fmicb.2022.990606 (PMC9577601; doi:10.3389/fmicb.2022.990606)
Supplement: Supplementary file 1 [file Data_Sheet_1.PDF]

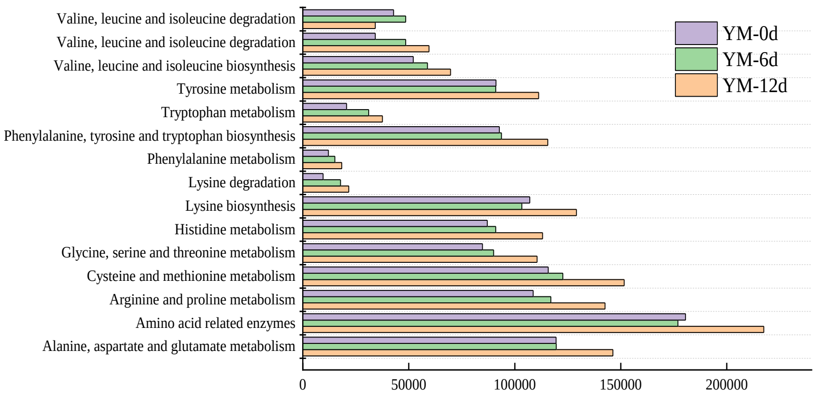

Fig. S1. Prediction of bacterial amino acid metabolism in fermented sausage

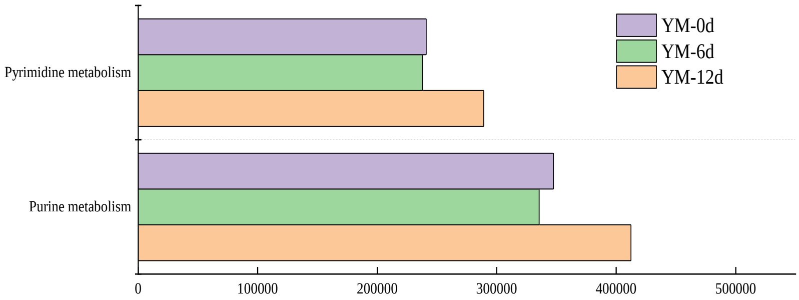

Fig. S2. Prediction of bacterial nucleotide metabolism in fermented sausage

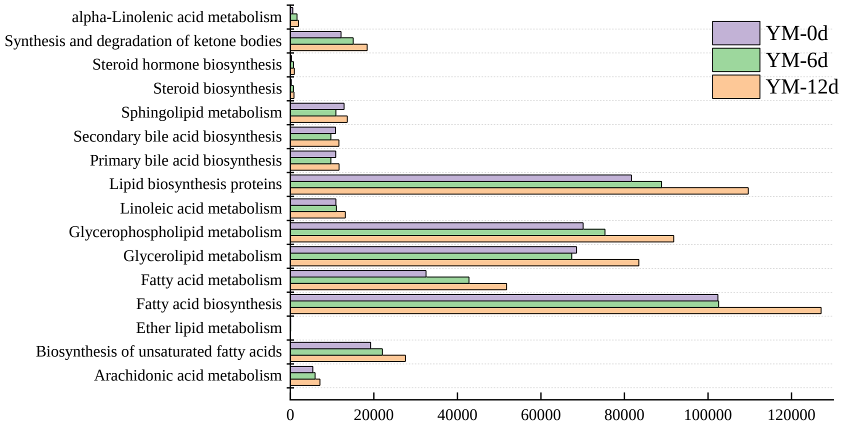

Fig. S3. Prediction of bacterial lipid metabolism in fermented sausage

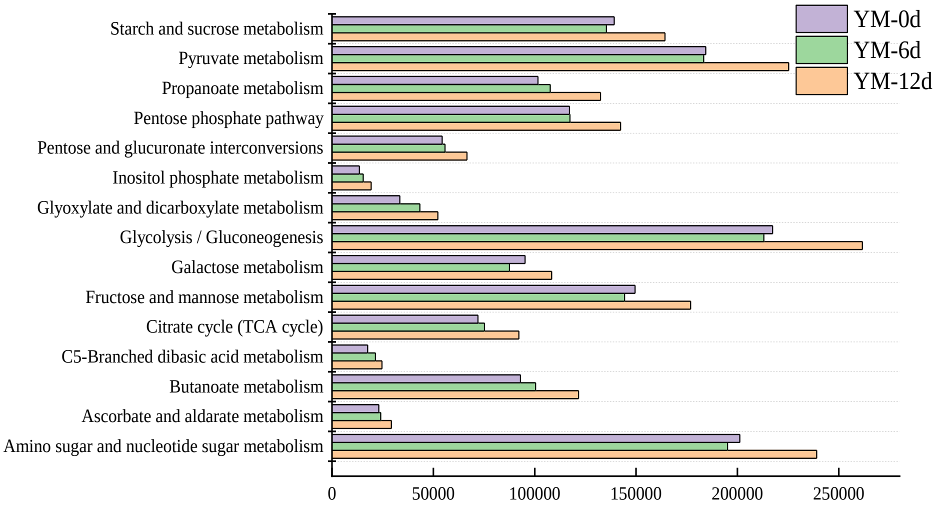

Fig. S4. Prediction of carbohydrate metabolism in sausage
